# ﻿Expanding the genus *Neobaryopsis* (Calcarisporiaceae, Hypocreales): descriptions of two new species and two new combinations based on morphological and molecular data

**DOI:** 10.3897/mycokeys.124.169315

**Published:** 2025-11-05

**Authors:** Valerii Darmostuk, Javier Etayo, Pamela Rodriguez-Flakus, Martin Kukwa, Adam Flakus

**Affiliations:** 1 W. Szafer Institute of Botany, Polish Academy of Sciences, Lubicz 46, PL-31-512 Krakow, Poland W. Szafer Institute of Botany, Polish Academy of Sciences Krakow Poland; 2 Navarro Villoslada 16, 3° dcha., 31003 Pamplona, Navarra, Spain Unaffiliated Navarra Spain; 3 Department of Plant Taxonomy and Nature Conservation, Faculty of Biology, University of Gdańsk, Wita Stwosza 59, PL-80-308 Gdańsk, Poland University of Gdańsk Gdańsk Poland

**Keywords:** Ascomycota, cloud forest, diversity, lichenicolous fungi, multi-locus phylogeny, South America, taxonomy

## Abstract

The genus *Neobaryopsis* (Calcarisporiaceae) comprises lichenicolous fungi with bright-colored perithecia, long, multiseptate, filiform ascospores, and mononematous conidiophores or distinctive synnematous-like asexual morphs. The genus was originally described from species growing on *Lobariella* spp., but its close morphological similarity to *Neobarya* long obscured its phylogenetic identity. In this study, we reassess the systematics of *Neobarya*-like taxa using a combination of morphological and multi-locus phylogenetic analyses (ITS, LSU, *tef1*, *rpb2*). Our results confirm that several lichenicolous species historically placed in *Neobarya* in fact belong to the genus *Neobaryopsis*, which is closely related to *Calcarisporium*. We describe two new species (*Neobaryopsis eriodermaticola* and *N. teloschistis*) and propose two new combinations (*Neobaryopsis ciliaris* and *N. peltigerae*). This work expands the genus *Neobaryopsis* to five recently accepted species, some of which exhibit strong host specificity and a primarily South American distribution. An identification key for species of *Neobaryopsis* and *Neobarya*-like fungi is provided. Our results clarify the systematics of these morphologically similar lichenicolous fungi and provide a framework for future studies on host specificity, biogeography, and evolutionary relationships within Hypocreales.

## ﻿Introduction

Lichenicolous fungi, a specialized ecological group associated with lichen hosts, represent a remarkably diverse assemblage with a wide range of morphological adaptations. They occur on all continents, but comprehensive studies have been limited to certain regions. In numerous cases, the lack of molecular sequence data for these fungi substantially limits the accuracy of their generic assignment and impedes confident inference of their phylogenetic placement. Recent studies have shown that the lichenicolous lifestyle has evolved independently multiple times throughout fungal evolutionary history. Moreover, this group also exhibits a high degree of morphological plasticity, making taxonomy based solely on morphology particularly challenging (Suija et al. 2015, 2024; Pino-Bodas et al. 2017; Flakus et al. 2019; Darmostuk and Flakus 2024; Diederich et al. 2024; Etayo et al. 2024).

The genus *Neobarya*, with the type species *Neobarya parasitica*, was established to accommodate fungicolous species with pyriform, light-colored (white, green, yellow, or orange) perithecia, lacking a distinct subiculum, and linear asci with long, septate, filiform ascospores (Eriksson and Hawksworth 1986). The generic concept was later expanded to include species growing on lichens that share similar morphological and anatomical features, although concerns were raised about the placement of fungicolous and lichenicolous taxa in the same genus (Etayo 2002; Candoussau et al. 2007; Etayo and Sancho 2008).

Lawrey et al. (2015) obtained molecular data for the generic type *Neobarya parasitica* and for the lichenicolous species *Neobarya usneae*. Their results showed that these two species belong to different families within Hypocreales, Clavicipitaceae and Hypocreaceae, respectively. Based on a combination of morphological features and phylogenetic evidence, the authors established a new lichenicolous genus, *Lichenobarya*, with *Lichenobarya usneae* as the type species.

Subsequently, Flakus et al. (2019) described another monotypic lichenicolous genus, *Neobaryopsis*, for *Neobarya*-like specimens growing on *Lobariella* spp. in Bolivia. This genus was defined by its narrowly pyriform, yellowish to orange ascomata; long, filiform ascospores; and a synnematous-like asexual morph with a white stipe and a hyaline, pink to yellowish-orange conidial mass. Recent phylogenetic analyses have shown that *Neobaryopsis* forms a well-supported clade sister to the anamorphic genus *Calcarisporium*, suggesting its affinity with the family Calcarisporiaceae, whereas *Neobarya* belongs to Clavicipitaceae (Flakus et al. 2019; Darmostuk et al. 2025).

Four additional *Neobarya* species growing on lichen hosts (*N. ciliaris*, *N. darwiniana*, *N. lichenophila*, and *N. peltigerae*) showed morphological similarity to *Neobaryopsis*, but for a long time molecular data for these taxa were lacking (Etayo 2002; Candoussau et al. 2007). In this study, we clarify the phylogenetic placement of *Neobarya ciliaris* and *N. peltigerae*, and we describe two additional species that belong to the genus *Neobaryopsis*.

## ﻿Material and methods

### ﻿Taxon sampling and morphological studies

This study is based on freshly collected material of lichenicolous species, complemented by herbarium specimens deposited at KRAM, LPB, UGDA, and the personal herbarium of J. Etayo (hb. Etayo). Morphological and anatomical characters were examined using standard stereo and compound microscopes (Nikon SMZ 800 and 80i DIC; Leica S9i and S9D). Ascomata sections were prepared manually using a razor blade or with a freezing sliding microtome Microm HM 430 (Thermo Fisher Scientific, USA) combined with a BFS-MP freezing stage and a BFS-3MP controller. Sections and squash mounts were examined in distilled water, 10% KOH (K), or lactophenol cotton blue (LPCB; Fluka, no. 61335-100ML). Photomicrographs of anatomical characters were obtained under transmitted differential interference contrast (DIC) microscopy. All measurements were made in distilled water or LPCB. Measurements (if n > 10) are given as (min.–)x̄–SD–x̄+SD(–max.), where x is the arithmetic mean and SD is the standard deviation.

### ﻿DNA extraction, PCR amplification, and DNA sequencing

Lichen thalli with ascomata of lichenicolous fungi were stored at –20 °C until processing. Ascomata were removed from the host thallus and carefully cleaned in double-distilled water on a microscope slide under sterile conditions to remove host tissues and other visible impurities using ultra-thin tweezers and a razor blade. DNA was extracted from 4 to 8 clean ascomata or hymenia, depending on each specimen, using the QIAamp DNA Investigator Kit (Qiagen, Germany) following the manufacturer’s instructions. Four loci — nuclear rDNA internal transcribed spacer (ITS), nuclear rDNA large subunit (LSU), a fragment of the DNA-directed RNA polymerase II subunit two gene (*rpb2*), and a fragment of the region coding for protein synthesis elongation factor 1 alpha (*tef1*) – were generated. The primers used and polymerase chain reaction (PCR) conditions are listed in Table 1. Amplification was performed in a total reaction volume of 25 μl, consisting of 3 μl (5 μl for protein-coding regions) of genomic DNA template, 1 μl (3 μl for protein-coding regions) of each forward and reverse primer, 13 μl of DreamTaq PCR Master Mix (2×) (Thermo Fisher Scientific, USA), and 7 μl (1 μl for protein-coding regions) of double-distilled, deionized water. PCR products were sequenced in both directions by Macrogen (Amsterdam, the Netherlands). The newly generated sequences were carefully checked, assembled, and edited manually using Geneious Pro 8.0 (Biomatters Ltd.) and were deposited in GenBank. Detailed information on the sequences used in this study is provided in Table 2.

**Table 1.** Loci, primers, and polymerase chain reaction (PCR) conditions used in this study.

Loci	PCR primers	Sequence (5′–3′)	PCR Cycles	References
ITS	ITS1F	CTT GGT CAT TTA GAG GAA GTA A	94 °C: 5 min (94 °C: 30 s, 54 °C: 30 s, 72 °C: 30 s) × 4 cycles; (94 °C: 30 s, 48 °C: 30s, 72 °C: 1 min) × 32 cycles. A final extension of 72 °C: 10 min	White et al. (1990); Gardes and Bruns (1993)
	ITS4	TCC TCC GCT TAT TGA TAT GC		
LSU	LROR	GTA CCC GCT GAA CTT AAG C	94 °C: 5 min (94 °C: 30 s, 54 °C: 30 s, 72 °C: 30 s) × 4 cycles; (94 °C: 30 s, 48 °C: 30s, 72 °C: 1 min) × 32 cycles. A final extension of 72 °C: 10 min	Rehner and Samuels (1994); Vilgalys and Hester (1990)
	LR5	TCC TGA GGG AAA CTT CG		
*tef1*	EF1-983F	GCY CCY GGH CAY CGT GAY TTY AT	(95 °C: 30 s, 55 °C: 50 s, 72 °C: 1 min) × 35 cycles. A final extension of 72 °C: 10 min	Rehner and Buckley (2005)
	EF1-2228R	AT GAC ACC RAC RGC RAC RGT YTG		
*rpb2*	fRPB2-5F	GAY GAY MGW GAT CAY TTY YGG	94 °C: 90 s (94 °C: 30 s, 55 °C: 90 s, 68 °C: 2 min) × 40 cycles. A final extension of 68 °C: 5 min	Liu et al. (1999)
	fRPB2-7CR	CCC ATR GCT TGY TTR CCC AT		

**Table 2.** List of specimens, with host/substrate, strain/voucher, country of origin, and GenBank accession numbers used in phylogenetic analyses.

Species	Strain / Voucher	Host / substrate	Country	GenBank accession numbers			
				ITS	LSU	tef1	rpb2
*Albomorchellophila morchellae*	KUNCC 21-10005 T	*Morchella* sp.	China	OP580900	OP580862	OP585424	–
*Albomorchellophila morchellae*	KUNCC 21-10100	*Morchella* sp.	China	OR420019	OR420021	OR420838	–
*Calcarisporium arbuscula*	CBS 111.57	*Russula fellea*	United Kingdom	MH857665	MH869205	–	–
*Calcarisporium arbuscula*	CBS 518.66	Boletus	the Netherlands	MH858872	MH870517	–	–
*Calcarisporium arbuscula*	CBS 900.68 T	decaying agaric	Germany	MH859249	KX442598	KX442596	KX442597
*Calcarisporium cordycipiticola*	CGMCC 3.17904	fruiting body of *Cordyceps militaris*	China	KT945001	KX442604	KX442605	KX442607
*Calcarisporium cordycipiticola*	CGMCC 3.17905 T	fruiting body of *Cordyceps militaris*	China	KT944999	KX442599	–	KX442594
*Calcarisporium cordycipiticola*	CGMCC 3.17906	fruiting body of *Cordyceps militaris*	China	–	KX442592	KX442591	KX442590
*Calcarisporium guizhouense*	DY05042	*Cordyceps* sp.	China	PP809658	PP809662	PP823899	–
*Calcarisporium guizhouense*	DY05041 T	*Cordyceps* sp.	China	PP124948	PP133530	PP146564	–
*Calcarisporium xylariicola*	HMAS 276836 T	*Xylaria* sp.	Italy	KX442603	KX442601	KX442595	KX442600
*Calcarisporium yuanyangense*	YFCC 22099256 T	*Ophiocordyceps nutans*	China	OQ954173	OQ954171	OQ981389	OQ981390
*Neobaryopsis andensis*	Etayo 20-11 (LPB)	*Lobariella pallida*	Bolivia	MT153958	MT153987	–	–
*Neobaryopsis andensis*	Flakus 25967.1 (KRAM L-71220.1) T	*Lobariella pallida*	Bolivia	MT153956	MT153985	PP583634	–
*Neobaryopsis andensis*	Flakus 25967.2 (KRAM L-71220.2)	*Lobariella pallida*	Bolivia	MT153957	MT153986	–	–
*Neobaryopsis ciliaris*	Kukwa 15174b (LPB)	*Leucodermia leucomelos*	Bolivia	**PX418470**	**PX418477**	**PX438769**	**PX438774**
*Neobaryopsis ciliaris*	Etayo 34451 (LPB)	*Leucodermia boryi*	Bolivia	**PX418469**	**PX418476**	**PX438768**	–
*Neobaryopsis eriodermaticola*	Kukwa s.n. (KRAM L-75209) T	*Erioderma* sp.	Bolivia	**PX418471**	**PX418478**	–	–
*Neobaryopsis eriodermaticola*	Etayo 33243 (LPB)	*Erioderma* sp.	Bolivia	**PX418472**	**PX418479**	**PX438770**	–
*Neobaryopsis peltigerae*	Stöckli s.n. (KRAM L-75209)	*Peltigera* sp.	Switzerland	**PX418473**	**PX418480**	**PX438771**	**PX438775**
*Neobaryopsis peltigerae*	Suija s.n. (TUF095161)	*Peltigera* sp.	Finland	**PX418483**	–	–	–
*Neobaryopsis peltigerae*	Darmostuk 1695 (KRAM L-75217)	*Peltigera* sp.	Norway	**PX418474**	**PX418481**	**PX438772**	**PX438776**
*Neobaryopsis teloschistis*	Kukwa 16418 (UGDA L) T	*Teloschistes flavicans*	Bolivia	**PX418475**	**PX418482**	**PX438773**	**PX438777**
*Verticimonosporium diffractum*	CBS 310.72 T	*Cocos nucifera*, decaying leaf	Papua New Guinea	MH860483	MH872200	–	–
*Verticimonosporium ellipticum*	CBS 100388 T	Palmae, decaying petiole	Peru	MH862700	MH874307	–	–

Sequences obtained in this study are shown in bold. Abbreviations: T: type specimen or ex-type strain; –: indicates unavailable data or sequence.

### ﻿Phylogenetic analyses

All generated sequences were first subjected to BLAST nucleotide searches (Altschul et al. 1990) to discard potential contamination by unrelated fungi. Alignments for each region were generated using MAFFT (Katoh and Standley 2013), as implemented on the GUIDANCE2 web server (Penn et al. 2010). The single-locus phylogenies for all loci were generated (results not shown) to detect incongruences in topology. PartitionFinder 2 (Lanfear et al. 2017) was used to select the best partition scheme for our dataset and substitution models for each partition. A single substitution model was selected for each region (K80+G for ITS1 and ITS2, TIM+I+G for 5.8S, LSU, the first codon position of *tef1* and *rpb2*, F81+I for the second codon position of *tef1* and *rpb2*, TVM+G for the third codon position of *tef1*, and HKY for the third codon position of *rpb2*) under a greedy search algorithm and the Akaike information criterion (AIC) (Lanfear et al. 2012). For *tef1* and *rpb2*, each codon position was analyzed as a distinct partition: the first, second, and third codon positions. The coding domain sequence (CDS) of the protein-coding regions was detected using the Augustus web tool (Stanke et al. 2008). The dataset included 21 specimens belonging to 12 species of the genera *Calcarisporium*, *Neobaryopsis*, and *Verticimonosporium* (Calcarisporiaceae), as well as two specimens of *Albomorchellophila morchellae*, which were selected as the outgroup.

Maximum likelihood (ML) analyses were carried out using a heuristic search as implemented in IQ-TREE 2.1.2 on CIPRES Science Gateway (Ronquist et al. 2012), and 1000 ultrafast bootstrap replicates were selected to estimate branch support (Minh et al. 2020). A Bayesian inference (BI) phylogenetic tree was generated in MrBayes 3.2.6 on CIPRES Science Gateway (Ronquist et al. 2012) using the partitions and substitution models obtained by PartitionFinder 2. The posterior probabilities were calculated by sampling trees using the Markov chain Monte Carlo (MCMC) approach. Two independent parallel runs were randomly started, each with four incrementally heated chains (the temperature parameter for MCMC chains was 0.15). The MCMC was allowed to run for 100 million generations, sampling every 1000^th^ tree and discarding the first 25% of the sampled trees as a burn-in factor. The analysis was stopped when the standard deviation of split frequencies dropped below 0.01. The resulting ML and BI phylogenetic trees were visualized in FigTree 1.4.4 (http://tree.bio.ed.ac.uk/software/figtree/) and Inkscape 0.92 (https://inkscape.org/). The final tree topology was based on the 50% majority-rule consensus tree from the BI analysis, with Bayesian posterior probabilities (BPP) and maximum likelihood bootstrap (MLB) values indicated above branches. Branches with Bayesian posterior probability values >0.97 and maximum likelihood bootstrap values >70 were considered supported.

## ﻿Results

### ﻿Phylogenetic analyses

**Figure 1.**
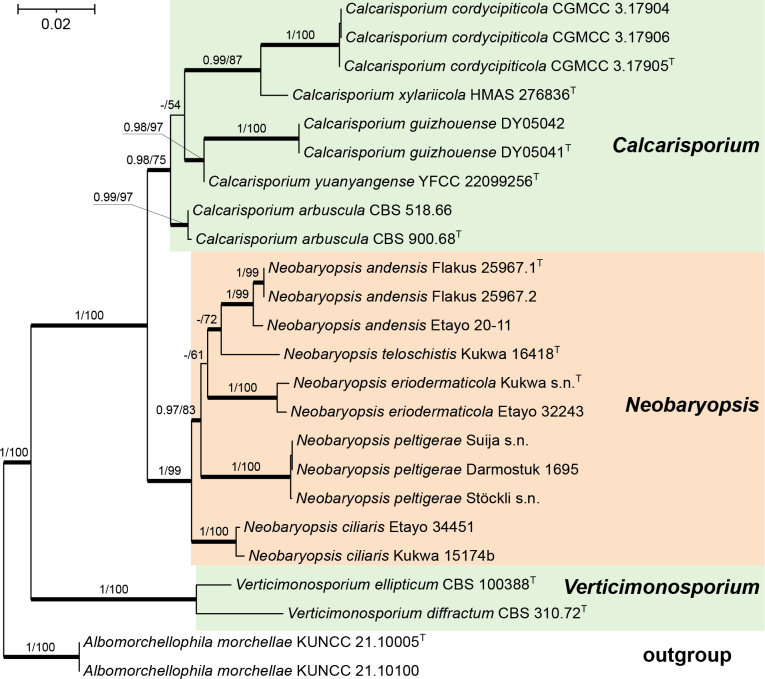
Phylogenetic relationships within the family Calcarisporiaceae inferred from a Bayesian Inference analysis (50% majority-rule consensus tree) of a combined ITS, LSU, *tef1*, and *rpb2* data set. Two specimens of *Albomorchellophila morchellae* were used as the outgroup. The numbers above branches indicate Bayesian posterior probabilities (BPP) > 0.97 and maximum likelihood bootstrap (MLB) support values > 70% respectively (BPP/ MLB). Sequences from type material are indicated by superscript T.

In this study, 25 new sequences from six specimens of lichenicolous fungi were generated. The multi-gene dataset of the family Calcarisporiaceae included 3221 characters (568 of ITS, 909 of LSU, 910 of *tef1*, and 834 of *rpb2*), of which 893 were parsimony-informative sites, 119 were singleton sites, and 1975 were constant sites. The phylogenetic tree showed a similar topology from ML and BI analyses; therefore, the BI tree was selected to represent and discuss the phylogenetic relationships among taxa (Fig. 1).

In our phylogenetic analyses, two species of the genus *Verticimonosporium* clustered in a well-supported clade (1/100) and showed a sister relationship to the remaining genera of Calcarisporiaceae. The five species of the anamorphic genus *Calcarisporium* included in the analyses clustered together in a moderately supported clade (0.98/75). The species *Neobarya ciliaris* and *N. peltigerae*, along with two undescribed lichenicolous species (growing on *Erioderma* sp. and *Teloschistes flavicans*) and *Neobaryopsis andensis*, formed a highly supported clade (1/99), which showed a sister relationship to the genus *Calcarisporium*. This clade comprises four highly supported subclades, corresponding to specimens from the same lichen hosts, as well as a singleton specimen on *Teloschistes flavicans*. However, the internal relationships within the *Neobaryopsis* clade remain unresolved. Two specimens of *Neobaryopsis eriodermaticola* clustered in a well-supported clade (1/100) and showed an unsupported sister relationship to the clade comprising *N. andensis* and *N. teloschistis*. The type species of *Neobarya*, *N. parasitica*, belongs to Clavicipitaceae and is phylogenetically unrelated to the genus *Neobaryopsis*.

### ﻿Taxonomy

#### ﻿ Neobaryopsis

Taxon classificationFungiHypocrealesCalcarisporiaceae

Flakus, Etayo, Kukwa & Rodr. Flakus

1BABD0C0-03E3-565F-9CE6-E5DD3B106C63

Neobaryopsis Flakus, Etayo, Kukwa & Rodr. Flakus, in Flakus, Etayo, Miadlikowska, Lutzoni, Kukwa, Matura & Rodriguez-Flakus, Plant and Fungal Systematics 64(2): 307 (2019)

##### Type species.

*Neobaryopsis andensis* Flakus, Etayo, Kukwa & Rodr. Flakus.

#### ﻿ Neobaryopsis ciliaris

Taxon classificationFungiHypocrealesCalcarisporiaceae

(Etayo) Darmostuk, Etayo, Kukwa & Flakus comb. nov.

165BF0BD-F123-58FE-9F15-B1B29B29D7BA

MycoBank No: 859618

Neobarya ciliaris Etayo, Biblioth. Lichenol. 84: 74 (2002). Basionym.

##### Typus.

Colombia • Nariño, Pasto, corregimiento El Encano, vereda Sta. Isabel, 30-S lago La Cocha (Guamués), 2700 m, en cilios de *Leucodermia lutescens*, July 1998, J. Etayo 17386, J. Muñoz & B. Ramírez (holotype COL!, isotype hb. Etayo!).

For detailed description see (Etayo 2002).

##### Notes.

This species was described from a few localities in Colombia growing on *Heterodermia* s. lat. (including *Leucodermia* spp.) (Etayo 2002), with subsequent records from Bolivia and Ecuador (Etayo 2017; this study). *Neobaryopsis ciliaris* typically grows on the cilia of the host, though in some specimens it was found on the lower surface of the thalli.

##### Specimens examined.

Bolivia • Dept. Chuquisaca, Prov. Belisario Boeto, close to Padilla between Nuevo Mundo and Santa Rosa, 18°57'12"S, 64°16'37"W, 1790 m, transition between Boliviano-Tucumano forests and dry inter-Andean vegetation, on cilia of *Leucodermia leucomelos*, 16 Jul. 2015, J. Etayo 29440 (hb. Etayo, LPB) • Prov. Hernando Siles, 15 km west of Monte Agudo, 19°48'57"S, 64°06'00"W, 1815 m, disturbed Tucumano-Boliviano forest, on cilia of *Leucodermia leucomelos*, 20 Jul. 2015, J. Etayo 30322 (hb. Etayo, LPB) • Dept. Cochabamba, Prov. Carrasco, near Río Batea Mayu close to Monte Punku, lower montane Yungas cloud forest, 17°32'27"S, 65°16'14"W, 2553 m, on corticolous *Leucodermia leucomelos*, 28 Nov. 2014, M. Kukwa 15158b (LPB) • ibid., 17°31'33"S, 65°16'21"W, 2430 m, lower montane Yungas cloud forest, on thallus of *Heterodermia* sp. on trunk, 28 Nov. 2014, J. Etayo 34196 (hb. Etayo) • ibid., near Río Lopez Mendoza, 17°30'25"S, 65°16'51"W, 2248 m, lower montane Yungas cloud forest, on thallus of *Heterodermia japonica* on trunk, 27 Nov. 2014, J. Etayo 33933 (LPB, hb. Etayo) • ibid., Wayra Mayu close to Monte Punku, 17°33'30"S, 65°16'08"W, 2750 m, on corticolous *Leucodermia leucomelos*, 28 Nov. 2014, M. Kukwa 15174b (LPB) • valle de Zongo, bosque nublado, near metal bridge, 16°07'41"S, 68°05'55"W, 2450 m, on cilia of *Heterodermia* sp., 29 May 2011, J. Etayo 26735 (LPB) • Prov. Tiraque, Parque Nacional Carrasco, Camino de las Nubes-Cotany Alto road, 17°17'28"S, 65°44'05"W, 4146 m, open high Andean vegetation, on cilia of corticolous *Leucodermia leucomelos*, 2 Dec. 2014, J. Etayo 29629 (LPB) • Dept. La Paz, Prov. Bautista-Saavedra, Área Natural de Manejo Integrado Nacional Apolobamba, near la Curva, W oriented valley close to Charazani, 15°06'30"S, 69°01'50"W, 3550 m, open area with schrubs and *Polylepis* near the river, on cilia of *Leucodermia boryi* on twigs, 14 Nov. 2014, J. Etayo 35009 (hb. Etayo, LPB) • Prov. Franz Tamayo, Área Natural de Manejo Integrado Nacional Apolobamba, below Pelechuco, 14°49'08"S, 69°03'50"W, 3560 m, open area with shrubs and Polylepis trees, on cilia of *Leucodermia boryi* on tree, 20 Nov. 2014, J. Etayo 34451 (hb. Etayo, LPB) • Parque Nacional y Área Natural de Manejo Integrado Madidi, below Keara Bajo, 14°41'47"S, 69°04'10"W, 3160 m, open area with shrubs and scattered trees, on cilia of *Leucodermia vulgaris* on trunk, 18 Nov. 2014, J. Etayo 34418 (hb. Etayo, LPB) • Dept. Santa Cruz, Prov. Caballero, El Camino de las Orquídeas, 17°50'27"S, 64°41'59"W, 2510 m, Yungas cloud forest, on cilia of *Heterodermia podocarpa*, 17 Aug. 2012, J. Etayo 28802 (hb. Etayo, LPB).

#### ﻿ Neobaryopsis eriodermaticola

Taxon classificationFungiHypocrealesCalcarisporiaceae

Darmostuk, Etayo, Kukwa & Flakus sp. nov.

8C766932-B37E-591B-8CF8-4E10EDA2730B

MycoBank No: 859616

Fig. 2

##### Typus.

Bolivia • Dept. Cochabamba, Prov. Chapare, Parque Nacional Carrasco, Sillar road close to Villa Tunari, 17°06'58"S, 65°41'19"W, 1020 m, Sub-Andean Amazon forest close to plantation, on apothecia of *Erioderma* sp., 3 Dec. 2014, M. Kukwa s.n. & A. Flakus (holotype KRAM L-75209, isotype LPB)

##### Etymology.

Named after the host lichen genus, *Erioderma*.

##### Description.

Ascomata perithecioid, in small groups, superficial, with loose white arachnoid subiculum, without stromata, pyriform to elongate pyriform, collapsing by lateral pinching when dry, (250–)310–370(–410) μm high, (160–)210–250(–300) μm wide (n = 15), smooth, pale orange to orange. Perithecial wall 25–40 µm thick, not changing color in K, composed of two regions: external, pale yellow region with thick-walled, isodiametric cells, 2–4 μm diam., and inner region with hyaline, thin-walled, flattened cells, 5–8 × 2.5–3.5 μm. Periphyses 0–1 septate, c. 7–10 × 1.5–3 µm. Asci cylindrical to narrowly cylindrical, 8-spored, (120–)135–160(–185) × (4.0–)4.2–4.8(–5.3) µm (n = 12), apex thickened with a conspicuous cap c. 3.5–4 µm high. Ascospores thread-like, multiseptate (septa hardly visible on young ascospores), not constricted at the septum, hyaline, with rounded apical parts, smooth-walled, twisted in the ascus, (80–)95–110(–120) × (1.8–)2.0–2.2(–2.4) µm (n = 20). Asexual stage hyphomycetous, present near the lower part of perithecia, colonies effuse. Conidiophores short, unbranched, 0–2-septate, hyaline. Conidiogenous cells terminal, hyaline, thin-walled, smooth, cylindrical to slightly tapering, phialidic, 25–40 × 1.5–2 μm. Conidia ellipsoidal to cylindrical, sometimes slightly constricted in the middle, hyaline, 0-septate, smooth, slightly truncated, (7.4–)8.0–9.4(–9.6) × (2.6–)2.8–3.8(–4.2) µm (n = 25).

**Figure 2.**
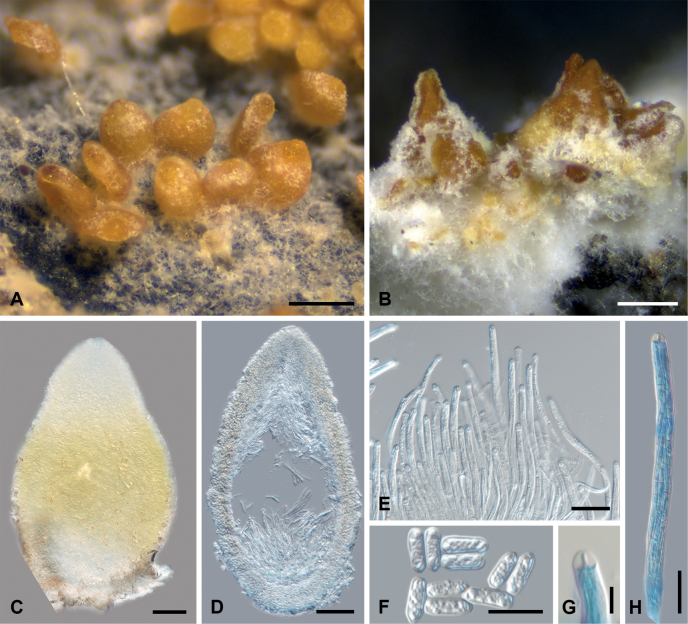
*Neobaryopsis eriodermaticola* (holotype, except B. and F. from Etayo 33243). A, B. Ascomata and white hyphomycetous colony on the host thallus; C. Squashed ascoma; D. Section of the ascoma; E. Asci; F. Conidia; G. Ascus apex (in LPCB); H. Ascus with ascospores. Scale bars: 250 µm (A, B), 50 µm (C, D), 25 µm (E), 10 µm (F, H), 5 µm (G).

##### Host, distribution, and ecology.

*Neobaryopsis eriodermaticola* is currently known from a few localities in the montane Yungas cloud forest in Bolivia (3200–3500 m) and the Sub-Andean Amazon forest, where it grows on apothecia or rarely on thallus of *Erioderma* spp.

##### Notes.

*Neobaryopsis eriodermaticola* is morphologically similar to *N. peltigerae*, which grows on *Peltigera* spp. (see notes below). *Neobaryopsis peltigerae* differs from the new species in possessing subglobose to slightly clavate terminal cells at the perithecial apex, a feature absent in *N. eriodermaticola*. Additionally, *N. peltigerae* has shorter ascospores, 35–75 µm (vs. (80–)95–110(–120) µm in *N. eriodermaticola*), and different lichen hosts (Candoussau et al. 2007). The anamorphic stage of *Neobaryopsis eriodermaticola* also has smaller conidia, (7.4–)8.0–9.4(–9.6) × (2.6–)2.8–3.8(–4.2) µm, compared to those reported from cultures of *N. peltigerae* (11–14 × 3–7 µm).

Our phylogenetic results indicate that the new species is sister to *Neobaryopsis andensis* and *N. teloschistis*, although this relationship lacks significant statistical support (Fig. 1). *Neobaryopsis andensis* can be distinguished by its larger ascomata, 500–700 µm high (vs. (250–)310–370(–410) μm high in *N. eriodermaticola*), longer asci and ascospores, smaller conidia, 4–7 × 2–2.5 μm (vs (7.4–)8.0–9.4(–9.6) × (2.6–)2.8–3.8(–4.2) µm in *N. eriodermaticola*), as well as different lichen hosts (Flakus et al. 2019).

##### Specimens examined.

Bolivia • Dept. Cochabamba, Prov. Tiraque, Parque Nacional Carrasco, Camino de los Nubes, Antenas Sillar-Villa Tunari old road, 17°12'32"S, 65°41'52"W, 3520 m, upper montane Youngas cloud forest, on thallus and especially apothecia of *Erioderma* sp. on twigs, 30 Nov. 2014, J. Etayo 33243 (hb. Etayo, LPB) • Dept. La Paz, Prov. Nor Yungas, Parque Nacional y Área Natural de Manejo Integrado Cotapata of Unduavi by Sillu Tincara pre-Columbian route, transition Páramo Yungeño – Yungas montane cloud forest, 16°17'22"S, 67°53'29"W, 3518 m, on *Erioderma leylandii*, 25 May 2011, J. Etayo 28016 (LPB) • ibid., bosque nublado yungas, sendero que parte de la Estación de servicio, 16°17'09"S, 67°51'00"W, 3220–3250 m, on *Erioderma leylandii* on twigs, 24 May 2011, J. Etayo 27692 (hb. Etayo).

#### ﻿ Neobaryopsis peltigerae

Taxon classificationFungiHypocrealesCalcarisporiaceae

(Lowen, Boqueras & Gómez-Bolea) Darmostuk comb. nov.

48E99CE4-FD0A-5DCD-8A9F-E913A67EEA46

MycoBank No: 859619

Neobarya peltigerae Lowen, Boqueras & Gómez-Bolea, Sydowia 59(2): 206 (2007). Basionym.

##### Typus.

Spain • País Valenciá, Pobla de Benifassá, barranc de l’Avellanar (serra dels Ports), BF 5408, elev. 1100 m, on *Peltigera membranacea*, 21 Oct. 1990, A. Gómez-Bolea & M. Boqueras (holotype NY 00921734 [photo!], isotype BCC-Lich 5239).

For detailed description see Candoussau et al. (2007). The ascospores in the examined specimens were thread-like, multiseptate (only ascospores with clearly visible septa were measured), not constricted at the septum, hyaline, with rounded apical parts, smooth-walled, twisted in the ascus, (100–)110–125(–130) × (1.5–)2.0–2.4(–2.8) µm (n = 18).

##### Notes.

This species was described as *Neobarya peltigerae* from Spain on *Peltigera membranacea* (Candoussau et al. 2007). It was later reported from scattered localities in Europe (Candoussau et al. 2007; Stenroos et al. 2016; Zimmermann and Berger 2018; Zimmermann and Feusi 2018; Westberg et al. 2022; Brackel and Wirth 2023; Mombert et al. 2023), Asia (Zhurbenko and Kobzeva 2014), and North America (Zhurbenko 2009).

The diagnosis of *Neobaryopsis peltigerae* indicates filiform, up to 15-septate ascospores measuring 130–185 × (4.5–)5.0–6.0(–6.5) µm, whereas the detailed description in the same work also provides measurements of smaller ascospores (Candoussau et al. 2007). The reported ascospore width in the diagnosis appears to be the widest among known species of the genus; however, it is not supported by our examination of the available material. Upon reviewing Fig. 12I, J in Candoussau et al. (2007: 207), we observed that the ascospore width is actually 1.5–2.5 µm. Based on this, we assume it is a typographical error in the protologue and suggest that the ascospore width of *Neobaryopsis peltigerae* should be interpreted as 1.5–2.5 µm.

The anamorphic stage was reported in cultures of *Neobaryopsis peltigerae* from the Scottish collection, but the culture is no longer accessible (Candoussau et al. 2007). Its anamorphic stage is characterized by monophialidic conidiogenous cells and hyaline, cylindrical conidia with a truncate base, 11–14 × 3–7 µm. It is similar to the known anamorphic stage of *Neobaryopsis andensis*, but the latter differs by much smaller, bacilliform-ellipsoidal conidia, 4–7 × 2–2.5 µm, as well as by a distinctive synnematous-like colony (Flakus et al. 2019).

##### Specimens examined.

Norway • Vestfold, Larvik Municipality, SW of Lysbu, Vemannsås reserve, 59°08'11"N, 9°57'17"E, 120 m, on *Peltigera* sp. on mosses, 21 Jul. 2025, V. Darmostuk 1695 (KRAM L-75217) • Switzerland, Lajoux, Jura, Les Embreux, on *Peltigera* sp., 16 Mar. 2023, E. Stöckli (KRAM L-75210).

#### ﻿ Neobaryopsis teloschistis

Taxon classificationFungiHypocrealesCalcarisporiaceae

Darmostuk, Etayo, Kukwa & Flakus sp. nov.

14BCB6DB-C6B7-5106-877C-44657E3C382A

MycoBank No: 859617

Fig. 3

##### Typus.

Bolivia • Dept. Chuquisaca, Prov. Luis Calvo, close to Ticucha, between Tranqua and Monte Agudo, Parque Nacional y Área Natural de Manejo Integrado Serranía del Iñao, 19°39'50"S, 63°49'14"W, 1022 m, on corticolous *Teloschistes flavicans*, 18 Jul. 2015, M. Kukwa 16418 (holotype UGDA, isotype LPB).

##### Etymology.

Named after the host lichen genus, *Teloschistes*.

##### Description.

Ascomata perithecioid, scattered, superficial, white loose arachnoid subiculum observed only at the lower part, pyriform to elongate pyriform, collapsing by lateral pinching when dry, (400–)480–530(–550) μm high, (190–)200–230(–270) μm wide (n = 12), smooth, orange, apical part bright orange. Perithecial wall 30–40 µm thick, not changing color in K, composed of two regions: external, yellow region with thick-walled, isodiametric cells, 2.5–3 μm diam., and inner region with hyaline, thin-walled, flattened cells, 3.5–10 × 2.5–4.0 μm. Asci cylindrical to narrowly cylindrical, 8-spored, (220–)260–300(–360) × (4.2–)4.4–5.0(–5.5) µm (n = 15), apex thickened with a conspicuous cap c. 3–4 µm high. Ascospores thread-like, multiseptate (septa hardly visible on young ascospores), not constricted at the septum, hyaline, with rounded apical parts, smooth-walled, twisted in the ascus, (220–)250–280(–300) × (1.2–)1.4–2.0(–2.2) µm (n = 20). Asexual stage hyphomycetous, present near the lower part of perithecia, colonies effuse. Conidiophores short, unbranched, 0–2-septate, hyaline. Conidiogenous cells terminal, hyaline, thin-walled, smooth, cylindrical to slightly tapering, phialidic, 20–30 × 1.5–2 μm. Conidia ellipsoidal, at one end wider, sometimes slightly constricted in the middle, hyaline, 0-septate, smooth, truncated, (8.4–)9.0–11.2(–11.6) × (3.2–)3.4–3.6(–4.8) µm (n = 30).

**Figure 3.**
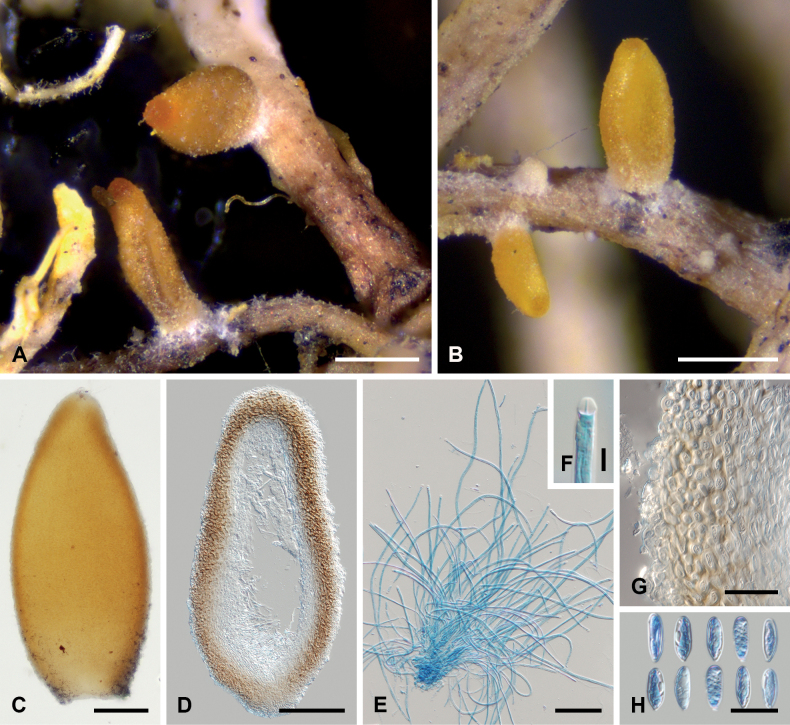
*Neobaryopsis teloschistis* (all from holotype). A, B. Ascomata on the host thallus; C. Squashed ascoma; D. Section of the ascoma; E. Asci; F. Ascus apex (in LPCB); G. Ascomatal wall; H. Conidia. Scale bars: 250 µm (A, B), 100 µm (C, D), 50 µm (E), 10 µm (G, H), 5 µm (F).

##### Host, distribution, and ecology.

*Neobaryopsis teloschistis* is currently known from a few localities in Bolivia, where it occurs on the thallus of *Teloschistes flavicans*. The fungal infection does not induce discoloration or necrosis of the host thallus. A specimen previously identified as *Nectria byssophila* on *Teloschistes flavicans* from Ecuador (Etayo 2017) likely represents *Neobaryopsis teloschistis*.

##### Notes.

This species is morphologically similar to *Neobaryopsis andensis*, but it can be distinguished by its scattered ascomata (400–)480–530(–550) µm (vs. aggregated ascomata 500–700 µm in *N. andensis*), narrower asci (4.2–)4.4–5.0(–5.5) µm wide (vs. asci 5–7 µm wide in *N. andensis*), and different lichen host (Flakus et al. 2019). Other species of the genus *Neobaryopsis* differ by smaller asci and ascospores, as well as their lichen hosts.

##### Specimens examined.

Bolivia • Dept. Tarija, Prov. Aniceto Arce, Papachacra, 21°41'52"S, 64°29'15"W, 1900 m, Tucumano-Boliviano altimontano forest, on *Teloschistes* sp., 8 Aug. 2012, J. Etayo 28243, 28350 (hb. Etayo, LPB).

## ﻿Discussion

In this study, we clarify the phylogenetic position of several lichenicolous fungi previously assigned to *Neobarya* and demonstrate the diversity of the genus *Neobaryopsis*. Multi-locus phylogenetic analyses revealed the placement of *Neobarya ciliaris* and *N. peltigerae* within *Neobaryopsis* (Calcarisporiaceae), distinct from *Neobarya* sensu stricto (Clavicipitaceae). These results confirm earlier assumptions, based on morphological and ecological data, that some lichenicolous species attributed to *Neobarya* had been placed in this genus only provisionally (Etayo 2002; Candoussau et al. 2007). Additionally, by describing *Neobaryopsis eriodermaticola* and *N. teloschistis*, which grow on *Erioderma* and *Teloschistes*, respectively, we expand the genus to include five currently known lichenicolous species.

The newly generated molecular data and resulting phylogenetic evidence further support the polyphyletic nature of so-called *Neobarya*-like fungi. This term has been used in several studies to refer to lichenicolous fungi typically characterized by sessile, bright-colored, pyriform perithecia, poorly developed interascal filaments, and long, septate, filiform ascospores (Etayo 2002, 2017; Etayo and Sancho 2008). Our findings are consistent with previous studies showing that morphologically similar yet phylogenetically unrelated species have been segregated from *Neobarya* into distinct genera, including *Neobaryopsis* (Calcarisporiaceae), *Lichenobarya* (Hypocreaceae), and *Rossmaniella* (Paranectriaceae) (Lawrey et al. 2015; Flakus et al. 2019; Darmostuk et al. 2025). These results support the idea that the lichenicolous lifestyle has evolved independently multiple times in distinct fungal lineages, as demonstrated, for example, in Acrospermales, Helotiales, Lecanorales, and Ostropales (Divakar et al. 2015; Suija et al. 2015; Pino-Bodas et al. 2017; Flakus et al. 2019; Darmostuk and Flakus 2024).

Despite the morphological similarity of the sexual stage in *Neobaryopsis*, we found considerable variation in the asexual stage. The generic type, *Neobaryopsis andensis*, produces a distinct synnematous-like asexual morph (Flakus et al. 2019). In contrast, most other examined species (*N. eriodermaticola*, *N. peltigerae*, and *N. teloschistis*) produce a hyphomycetous asexual morph with effuse mononematous conidiophores. Nonetheless, the conidiogenous cells and conidia are morphologically similar across the genus. Differences in colony structure of the asexual stage may reflect adaptations to particular microenvironmental conditions or host-specific factors. Such patterns, in which fungi adapt their morphology in response to microenvironmental conditions, are mostly known for pathogenic or saprotrophic fungi (Nadal et al. 2008; Wang and Lin 2012; Lin et al. 2015; Francisco et al. 2019), but this still needs to be studied in lichen-inhabiting fungi.

Lichenicolous fungi as an ecological group often exhibit varying degrees of host specificity. It can vary considerably and often remains uncertain in several less-studied groups, reflecting differences in the quality of both fungal and host taxonomy, as well as the availability of molecular and distributional data (Diederich et al. 2018). The genus *Neobaryopsis* appears largely host-specific: *N. andensis* occurs exclusively on *Lobariella* species, *N. eriodermaticola* on *Erioderma*, *N. teloschistis* on *Teloschistes*, and *N. peltigerae* on *Peltigera*. Only *N. ciliaris* shows a broader host range, occurring on several species of *Heterodermia* and *Leucodermia* (both belonging to the Physciaceae), which likely reflects the comparatively well-documented distribution of the species (Etayo 2002, 2017). Nonetheless, host specificity in *Neobaryopsis* should be re-evaluated with larger sampling, as most species are known from only a few collections.

Species of *Neobaryopsis* are recorded predominantly from South America (Bolivia, Colombia, and Ecuador), particularly in the Andean Yungas forests (from montane to cloud forests), with *N. peltigerae* also known from Europe, Asia, and North America (Etayo 2002, 2017; Westberg et al. 2022; Brackel and Wirth 2023; Mombert et al. 2023). However, the available distributional data remain extremely limited, making it impossible to determine whether current patterns reflect the diversification history of the genus, with a potential diversity hotspot in the Neotropics, or simply result from under-sampling in other tropical and extratropical regions.

Our phylogenetic results show that species of *Neobaryopsis* form a strongly supported clade at the generic level, although relationships among species remain unresolved. This may be due to uneven representation of sequence data and insufficient overall sampling. *Neobaryopsis teloschistis* is represented by a single sequenced specimen and shows a moderately supported sister relationship to *N. andensis*. While the phylogenetic position of *N. teloschistis* requires further confirmation, morphological features and host association strongly support its recognition as a distinct species. Unfortunately, we were unable to obtain molecular data for two additional lichenicolous species currently placed in *Neobarya*, *Neobarya darwiniana* and *N. lichenophila*. *Neobarya darwiniana* was described from *Nephroma antarcticum* in Chile, and no recent collections are available for molecular analysis (Etayo and Sancho 2008). *Neobarya lichenophila* is known only from its type collection and is morphologically strongly distinct from the rest of *Neobaryopsis* (Ferdinandsen and Winge 1909; Candoussau et al. 2007). Notably, the ascomata are dark-colored, turning orange in KOH, and the ostiolar region is composed of a palisade of hyphal elements — features not observed in any of the *Neobarya*-like genera. Thus, the current placement of *Neobarya darwiniana* and *N. lichenophila* remains uncertain and will require additional collections and molecular data for clarification.

Another *Neobarya*-like genus, *Leptobarya*, is characterized by orange to black, pyriform perithecia, abundant paraphyses, a thin-walled apical ascus region, and ascospores that disarticulate within the ascus (Etayo 2017). The genus includes two species known from South America. However, molecular data are currently lacking for both, and their phylogenetic relationships to other *Neobarya*-like genera remain unresolved.

### ﻿Key to species of *Neobaryopsis* and lichenicolous species of *Neobarya*

**Table:** 

1	Ascomata dark-colored when dry, ostiolar cap is formed of a palisade of hyphal elements; growing on *Cladonia*	***Neobarya lichenophila***
–	Ascomata yellow to orange when dry, ostiolar cap does not form a palisade of hyphal elements; growing on different hosts	**2**
2	Ascomata 170–200 µm high and 70–100 µm wide; ascospores 46–66 × 1–1.5 µm; growing on *Nephroma antarcticum*	***Neobarya darwiniana***
–	Ascomata and ascospores bigger; growing on different hosts	**3**
3	Ascomata 500–700 µm high and 250–300 µm wide; ascospores 225–430 × 1.5–2.5 µm; growing on *Lobariella*	***Neobaryopsis andensis***
–	Ascomata and ascospores smaller; growing on different hosts	**4**
4	Ascomata 400–600 µm high; growing on *Erioderma* spp. or *Heterodermia* s.lat.	**5**
–	Ascomata 250–480 µm high; growing on *Peltigera* spp. or *Teloschistes* spp.	**6**
5	Ascomata 400–600 µm high and 120–180 µm wide; ascospores 140–190 × 0.8 µm; growing on *Heterodermia* s.lat. (incl. *Leucodermia*)	***Neobaryopsis ciliaris***
–	Ascomata 400–550 µm high and 190–270 µm wide; ascospores 220–300 × 1.2–2.2 µm; growing on *Teloschistes* spp.	***Neobaryopsis teloschistis***
5	Ascomata 260–480 µm high and 160–360 µm wide; ascospores 130–185 × 1.5–2.5 µm; growing on *Peltigera* spp.	***Neobaryopsis peltigerae***
–	Ascomata 250–410 µm high and 160–300 µm wide; ascospores 80–120 × 1.8–2.4 µm; growing on *Erioderma* spp.	***Neobaryopsis eriodermaticola***

## Supplementary Material

XML Treatment for Neobaryopsis

XML Treatment for Neobaryopsis ciliaris

XML Treatment for Neobaryopsis eriodermaticola

XML Treatment for Neobaryopsis peltigerae

XML Treatment for Neobaryopsis teloschistis

## ﻿Additional information

### Use of AI

No use of AI was reported.

### Author ORCIDs

Valerii Darmostuk https://orcid.org/0000-0003-1430-1755

Javier Etayo https://orcid.org/0000-0003-0392-0710

Pamela Rodriguez-Flakus https://orcid.org/0000-0001-8300-5613

Martin Kukwa https://orcid.org/0000-0003-1560-909X

Adam Flakus https://orcid.org/0000-0002-0712-0529

### Data availability

All of the data that support the findings of this study are available in the main text.
